# The effect of adipose-derived mesenchymal stem cells against high fructose diet induced liver dysfunction and dysbiosis

**DOI:** 10.1007/s00210-024-03518-5

**Published:** 2024-11-05

**Authors:** Marwa Abdeltawab Mohammed, Nesma Hussein Abel Hay, Maha Tarek Mohammed, Hoda Sayed Mahmoud, Manar Yehia Ahmed, Ahmed Abdelmenem, Dina Sayed Abdelrahim

**Affiliations:** 1 Department of Physiology, Faculty of Medicine, Beni-Suef University, Beni-Suef, Egypt; 2 Department of Medical Biochemistry and Molecular Biology, Faculty of Medicine, Ain Shams University, Cairo, Egypt; 3 Department of Clinical Pharmacology, Faculty of Medicine, Ain Shams University, Cairo, Egypt; 4 Department of Forensic Medicine and Clinical Toxicology, Faculty of Medicine, Beni-Suef University, Beni-Suef, Egypt; 5 Department of Pharmacology, Faculty of Medicine, Modern University for Technology and Information, Cairo, Egypt

**Keywords:** Mesenchymal stem cells, SREBP-1, NOX4, MALAT-1

## Abstract

High fructose diet (HFrD) has been approved to be involved in the pathogenesis of insulin resistance. Mesenchymal stem cells have a vital role in the treatment of various diseases including metabolic disturbances. We investigated the effect of Adipose-derived mesenchymal stem cells (ADMSCs) against HFrD-induced metabolic disorders and the molecular mechanisms for this effect. Rats were divided into 3 groups; control, HFrD, and combined HFrD with ADMSCs. We assessed liver functions, gut microbiota activity, oxidative stress, adiponectin, and IL10 levels. Also, we measured SREBP-1, IRS-1 expression using Western blot, and Malat1 expression using rt-PCR. ADMSCs antagonized metabolic abnormalities induced by HFrD in the form of improvement of liver functions and alleviation of oxidative stress. In addition, ADMSCs ameliorated gut microbiota activity besides the elevation of adiponectin and IL10 levels. ADMSCs attenuated insulin resistance through upregulation of IRS1 and downregulation of SREBP-1 and Malat1. ADMSCs can protect against HFrD-induced metabolic hazards.

## Introduction

Increased fructose consumption has been linked to several health issues, including insulin resistance and liver dysfunction. Many artificial foods and beverages contain high amounts of fructose. The metabolism of fructose is mostly dependent on the liver. On the other hand, consuming too much fructose can upset the liver’s metabolic processes, which leads to the accumulation of a lot of fat and consequently development of fatty liver.

In addition, elevated fructose has the potential to disrupt glucose metabolism and throw off insulin signaling pathways. The mechanisms behind the development of metabolic disorders may be worrisome if they involve aberrant glucose synthesis, inflammation, or malfunctioning adipose tissues (Ouyang et al. 2008). Furthermore, a high fructose diet contributes to the development of liver dysfunction through the induction of oxidative stress as fructose was found to decrease the levels of GPx and SOD (Park et al. 2020). Also, fructose prompts the establishment of inflammation via activation of IL-1β, IL-6, and TNF-α plus a reduction of IL-10 (Cheng et al. 2022).

Stem cells are multipotent, undifferentiated cells that can differentiate into various cell types. They possess strong immunomodulatory and anti-inflammatory properties. Additionally, they can enhance insulin sensitivity. Several studies have demonstrated the role of stem cells in mitigating liver diseases and insulin resistance (Aziz et al. 2007; Diaz-Diaz et al. 2015). ADSCs have been preferred over other stem cells as studies revealed that ADSCs have genetic and epigenetic stability (Ra et al. 2011) and no serious immune response was detected with ADSCs use (Arnalich-Montiel et al. 2008; Yan et al. 2024).

Concerning the antioxidant properties of ADMSCs, it was demonstrated that ADMSCs inhibited ROS by means of a decrease of MDA besides, an increase of GSH, SOD, and TAC in diabetic rats (El-Sawah et al. 2022).

The colon is home to a vast variety of germs that are extremely harmful to health. In fact, these bacteria not only regulate the function of the gut barrier but also secrete tiny particles and compounds with antioxidant and anti-inflammatory properties. Conversely, other microbiota metabolites might be harmful (Moran-Ramos et al. 2017). Therefore, microbiota plays a crucial role in maintaining physiological functioning, and any disruption causes organ dysfunction and accelerates the development of metabolic diseases (Wang et al. 2020).

From the beneficial metabolites, short-chain fatty acids (SCFAs) affect the regulation of the epithelial gut barrier and permeability by modulating the production of tight junction proteins (Macia et al. 2015). This action is highly important to avoid entering pathogenic bacteria-produced toxic materials into the circulation. On the other hand, another metabolic product for microbiota, Lipopolysaccharides, collaborates with inflammation and metabolic issues (Cani et al. 2007; Mehta et al. 2010).

Metastasis-associated lung carcinoma transcript-1 (MALAT1) is a long noncoding RNA that plays an important role in the regulation of different physiological functions. MALAT1 is greatly involved in abnormal fatty acid synthesis resulting in lipid accumulation (Lu et al. 2022). In addition, MALAT1 stimulates the expression of sterol regulatory element-binding protein-1 (SREBP-1c) which is a vital member in the regulation of cholesterol and fatty acid synthesis in fatty liver disease and other fat metabolism disorders (Yan et al. 2016).

Therefore, in this study, we supposed that ADMSCs can ameliorate the hazards induced by a high fructose diet, and we implied the molecular mechanisms for this protection.

## Material and methods

### Experimental animals

Thirty male Wistar rats, weighing 160–180 gm, were purchased from the Holding Company for Biological Products and Vaccines (VACCERA), Helwan, Egypt. The animals were housed in the Clinical Pharmacology Department, Faculty of Medicine, Ain Shams University, Cairo, Egypt. Rats were allowed to acclimatize to the laboratory conditions for 2 weeks with a balanced rat chow diet (Meladco, El Obour, Egypt) and tap water available ad libitum. The animals were maintained at 25 ± 5 °C, relative humidity of 50 ± 10%, and 12 h light/dark cycle. The present study was conducted under the ethical standards of the Ain Shams University Research Committee the application approval number is FWA 000017585.

### Adipose-derived mesenchymal stem cells (ADMSCs) preparation

Adipose tissues from the rats’ abdomen walls were taken and placed into a sterile tube holding 15 mL of a phosphate-buffered solution (PBS; Gibco/Invitrogen, Grand Island, NY, USA). Using 0.075% collagenase II, enzymatic digestion was carried out in Hank’s Balanced Salt Solution with shaking at 37 °C for 60 min (SERVA Electrophoresis GmbH, Heidelberg, Germany). Digested tissue was then filtered and centrifuged, while erythrocytes were extracted using an erythrocyte lysis solution. The cells were delivered to tissue culture flasks containing 10% fetal bovine serum (FBS) (Gibco/BRL) with Dulbecco’s modified Eagle’s medium (DMEM, Gibco/BRL, Grand Island, NY, USA). Following a 24-h attachment period, non-adherent cells were eliminated by a PBS wash. Following incubation in DMEM supplemented with 10% FBS, 1% penicillin–streptomycin (Gibco/BRL), and 1.25 mg/L amphotericin B (Gibco/BRL), the attached cells were grown in vitro. Following two PBS washes at 80–90% confluence, 0.25% trypsin was added to 1 mM EDTA (Gibco/BRL) and allowed to sit for 5 min at room temperature. After centrifugation, the cells were placed back into a medium that contained serum. The cells were centrifuged and then re-infused with a serum-containing media. After that, they were incubated in a Falcon brand culture flask of 50 cm^2^. These cultures were considered first-passage cultures. The fusiform shape and adhesiveness of the cells were used to identify them as mesenchymal stem cells. This was confirmed by analyzing the CD markers using flow cytometry and fluorescent analysis cell sorting (FACS analysis). Cells were re-suspended in wash buffer after a brief centrifugation, (BD Biosciences, Germany). Three hundred milliliters of cell suspension was incubated with antibodies against CD29, CD34, and CD90 conjugated with Allophycocyanin (APC), Cyanine 5 (CY5), phycoerythrin (PE), and fluorescein isothiocyanate (FITC) dyes respectively for 45 min. Flow cytometry was performed on a FACS Calibur (BD Biosciences, Germany), and Cell Quest software was used for analysis.

### Experimental groups

Rats were randomly assigned to three groups, with 10 rats in each group: The control group: was fed a balanced chow diet purchased from Miladco for the Poultry Forage Industry, in Al-Obour City, Cairo, Egypt. The diet consisted of 48.8% carbohydrates, 21% protein, 3% fat, 0.8% calcium, 0.4% phosphorus, 5% fiber, 13% moisture, and 8% ash, with a total energy content of approximately 306.2 kcal per 100 g. The animals were fed ad libitum for 8 weeks before being examined. The high fructose diet group (HFrD): received oral fructose (Uni Fructose® Powder, 450 g, Universal Pharmaceutical Industries Company, Al-Obour City, Cairo, Egypt), dissolved in drinking water at a concentration of 60% fructose (w/v) for 8 weeks (Ushio et al. 2013). The combined group: received fructose in the same manner as the HFrD group but also received intravenous injections of a single dose of 1 ml of ADMSCs (3 × 10^6^ cells/ml) after 4 weeks of fructose administration (Ahmed et al. 2021).

### Assessment of body weight change

Rats’ body weights from different studied groups were measured weekly to assess the changes in their weight.

### Sacrifice and sample collection

On the day of sacrifice, rats were anesthetized with an intraperitoneal injection of Pentobarbitone (40 mg/kg body weight) with booster doses as needed. The dose was given according to the guidelines of rodent anesthesia analgesia formulary-UBC animal care (Flecknell et al. 2015). Blood samples were collected from the animals’ tail veins. After blood collection, rats were euthanized, and liver and adipose tissue samples were collected.

### Biochemical assay

#### Measurements of serum triglyceride (TAG) and total cholesterol

Measurement of TAG using Triglyceride Quantification Colorimetric/Fluorometric Kit (Biovision, USA, Catalog # K622-100) and the colored product of the reaction was measured using spectrophotometer at wavelength = 570 nm, and total cholesterol by QuickDetectTM Total cholesterol Rat ELISA Kit (Biovision, USA Catalog # K4436-100) was performed. The procedures were performed according to the manufacturer’s instructions.

#### Measurements of liver enzymes (ALT and AST) in serum samples

The serum levels of alanine aminotransferase (ALT) and aspartate aminotransferase (AST) were respectively measured using Alanine Aminotransferase rat ELISA Kit (Biomatik, Canada, Catalog # EKU02211) and Rat Aspartate Aminotransferase ELISA Kit (Biomatik, Canada, Catalog # EKE62019) according to manufacturer’s instructions.

#### Measurements of fasting glucose and adiponectin (ADP) in serum samples

Fasting glucose was measured using an enzymatic colorimetric Kit (Spectrum, Germany, catalog # 250001), and the colored product of the reaction was measured using a spectrophotometer at wavelength = 546 nm. Adiponectin was measured by Rat Adiponectin ELISA Kit (Cusabio, USA, Catalog # CSB E07271r) according to the manufacturer’s instructions.

#### Measurements of serum lipopolysaccharides (LPS) and short-chain fatty acid (SCFA)

LPS level was measured using LPS ELISA Kit (Antibodies, USA, Catalog # ABIN1169646). SCFA was measured using SCFA ELISA Kit (SunLong, China, Catalogue # SL1669Ra).

#### RNA extraction and RT-qPCR real-time quantitative PCR (RT-qPCR) was employed to detect MALAT-1 expression in liver tissues

Following the manufacturer's instructions, RNA was extracted using a nucleic acid extraction kit (NucleoSpin® REF.740901.250) that was purchased from Macherey–Nagel GmbH & Co. KG-Germany. Spectrophotometry was used to determine the concentration and purity (A260/A280 ratio) of RNA (dual wavelength Beckman, Spectrophotometer, USA). For later usage, the extracted and purified RNA samples were kept in storage at − 80 °C.

#### Quantitative real-time–polymerase chain reaction (RT-qPCR) assessment

The kit was supplied by Bioline, a median life science company in the UK (SensiFASTTM SYBR® Hi-ROX One-Step Kit, catalog #.PI-50217 V). The target genes (MALAT1) under investigation were forward 5′-GCCATTCCAGGTGGTGGTATTTAG-3′ and reverse 5 ~ -CAGATTCTGTGTTATGCCTGGTTAG-3′ primer sequences (gene bank accession number NR_144568.1). and primers sequences for reference housekeeping gene *(GAPDH*) were forward 5′-ATCGTGCGGGACATCAA-3′, and reverse 5′-AGGAAGGAGGGCTGGAA (gene bank accession number NM_001394060.2). The prepared reaction mix samples were applied in real-time PCR (StepOne Applied Biosystem, Foster City, USA). SensiFAST™ SYBR® Hi-ROX One-Step Kit was compatible with three-step cycling as seen in Table 1.

**Table 1:** The thermal profile cycling of qRT-PCR

Cycles	Temp	Time	Notes
1	45 °C	10 min	Reverse transcription
1	95 °C	2 min	Polymerase activation
40	95 °C 60 °C 72 °C	5 s 10 s 5 s	Denaturation Annealing Extension (acquire at end of step)

#### Calculation of relative quantification (RQ) (relative expression)

After the RT-PCR run the data were expressed in Cycle threshold (Ct). The PCR data sheet includes Ct values of the assessed gene MALAT and the housekeeping (reference) gene GAPDH). In order to measure the gene expression of certain genes, a control sample should be used. The relative quantitation (RQ) of each target gene is quantified according to the calculation of delta-delta Ct (ΔΔCt). We calculated the RQ of each gene by taking 2^−∆∆Ct^ as follows: ΔΔCt = [(Ct target, Sample) − (Ct ref, Sample)] − [(Ct target, Control)- (Ct ref, Control)].

#### Preparation of tissue homogenate and protein determination

Ten percent tissue homogenate was made at 40 °C in 0.05 M phosphate buffer (pH 7), using a polytron homogenizer. To eliminate mitochondria, erythrocytes, nuclei, and broken cells, the homogenate was centrifuged for 20 min at 10,000 rpm. Using a protein estimation kit (Bangalore, Indian, Catalog #2,624,800,021,730), the protein content of the tissue was determined (Bradford 1976).

ELISA kit was measured using an ELISA reader. An Enzyme-Linked Immuno-Sorbent Assay (ELISA) plate reader (Stat Fax 2200, Awareness Technologies, FL, USA) was used to measure color absorbance between 490 and 630 nm.

#### Measurement of proinflammatory cytokines and oxidative stress markers in liver and adipose tissues by enzyme-linked immunosorbent assay ELISA

Using commercial ELISA kits (Cloud Clone Corp., USA, Catalog #SEA056Ra), the levels of interleukin-10 (pg/mg) were estimated. In accordance with the protocol of the manufacturer’s manual, the activities of SOD (U/mg) and NOX4 (ng/mg) were assessed by using superoxide dismutase (SOD) Activity Assay Kit (BioVision, USA, Catalog #K335-100) and the colored product of the reaction was measured using spectrophotometer at wavelength = 450 nm, and Aviva Systems Biology’s NADPH oxidase (NOX4) ELISA Kit Catalog #OKEH01057) respectively.

#### Western blot was performed to determine the protein levels of hepatic SREBP-1c, IRS-1, and AMPK

Following the manufacturer's instructions, each homogenized tissue sample was combined with the ReadyPrepTM protein extraction kit (total protein) from Bio-Rad Inc. (Catalog #163–2086). Following the manufacturer’s instructions supplied by Bio Basic Inc. (Markham, Ontario, L3R 8T4 Canada), a Bradford Protein Assay Kit (SK3041) for quantitative protein analysis was utilized to determine the protein content in each sample. At a protein concentration of 20 μg, each sample was mixed with an equal volume of 2 × Laemmli sample buffer, which contained 4% SDS, 10% 2-mercaptoethanol, 20% glycerol, 0.004% bromophenol blue, and 0.125 M Tris HCl. After measuring, the pH was detected to be 6.8. Each previous mixture was heated for 5 min at 95 °C before being loaded onto a polyacrylamide gel electrophoresis to attain protein denaturation. Polyacrylamide gels were made using the TGX Stain-Free™ FastCast™ Acrylamide Kit (SDS-PAGE) supplied by Bio-Rad Laboratories (Inc Cat # 161–0181) and prepared according to the manufacturer’s instructions. The gel was put together in a transfer sandwich including (filter paper, a PVDF membrane, the gel, and the filter paper) going from bottom to top. The sandwich was placed in a transfer tank containing 1 × transfer buffer (25 mM Tris, 190 mM glycine, and 20% methanol). The blot was then performed at 25 V for 7 min to allow protein bands to transfer from gel to membrane using the BioRad Trans-Blot Turbo. The membrane was blocked for 1 h at room temperature in tris-buffered saline with Tween 20 (TBST) buffer and 3% bovine serum albumin (BSA). The blocking buffer’s components were the following: 150 mM NaCl, 20 mM tris (pH 7.5), 0.1% TBST, and 3% BSA. We purchased the primary antibodies for AMPK (Cell Signalling Technology, cat#2532), IRS-1 (Santa Cruz Biotechnology, Inc., USA, cat# sc-8038), and SREBP-1 (Santa Cruz Biotechnology, Inc., USA, cat# sc-365513). Primary antibodies were diluted in TBST (1:500) as directed by the manufacturer. Each primary antibody solution was incubated at 4 °C overnight with the blotted protein of interest. The blot was washed with TBST 3–5 times for 5 min. For 1 h at room temperature, the blotted target protein was incubated in the HRP-conjugated secondary antibody solution (Goat anti-rabbit IgG-HRP-1mg Goat mab -Novus Biologicals). With TBST, the blot was washed 3–5 times for 5 min. As instructed by the manufacturer, the chemiluminescent substrate (Clarity TM Western ECL substrate, Bio-Rad cat#170–5060) was applied to the blot. In brief, equal amounts of solution A (Clarity Western luminal/enhancer solution) and solution B (peroxidase solution) were added. A CCD camera-based imager was used for capturing the chemiluminescent signals. By protein normalization on the ChemiDoc MP imager, the band intensity of the target proteins was read against beta-actin (housekeeping protein) in the control sample using image analysis software.

### Statistical analysis

All values were presented as mean ± SD for each group. Statistical analysis was carried out using GraphPad Prism, software program, version 9.0 (2020) Inc., CA, USA. Data was tested for normality using D’Agostino and Person omnibus normality test. Statistical difference among groups was determined using a two-way analysis of variance (ANOVA) followed by Tukey’s multiple comparison test. *P* values < 0.05 will be considered statistically significant.

## Results

### Effect of ADMSCs treatment on body weight

**Fig. 1:**
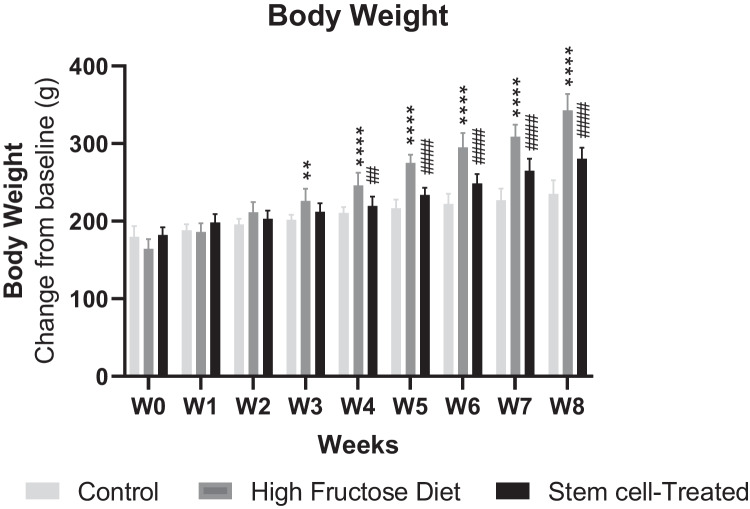
Effect of MSCs treatment on body weight of HFrD rat model: data are mean ± S.D. *n* = 10 rats per group. ^**^P < 0.01 vs. control group, ^****^P < 0.0001 vs. control group, ^##^*P* < 0.01 vs. HFrD group, ^####^*P* < 0.0001 vs. HFrD group by one-way ANOVA with Tukey’s multiple comparison test. MSCs: mesenchymal stem cells, HFrD: high fructose diet

As shown in Fig. 1, rats on HFrD showed a significant increase in body weight compared to control rats at the end of the 3rd (*p* < 0.01), 4th (*p* < 0.0001), 5th (*p* < 0.0001), 6th, (*p* < 0.0001), 7th (*p* < 0.0001) and 8th (*p* < 0.0001) weeks by 1.46 times at the end of the 8th week. On the other hand, ADMSCs treatment caused a significant reduction in body weight at the end of the 4th (*p* < 0.01), 5th (*p* < 0.0001), 6th, (*p* < 0.0001), 7th (*p* < 0.0001), and 8th (*p* < 0.0001) weeks with percent change 22.39% compared to HFrD- fed rats at the end of the 8th week (Fig. 1).

### Effect of ADMSCs treatment on fasting serum glucose level

**Fig. 2:**
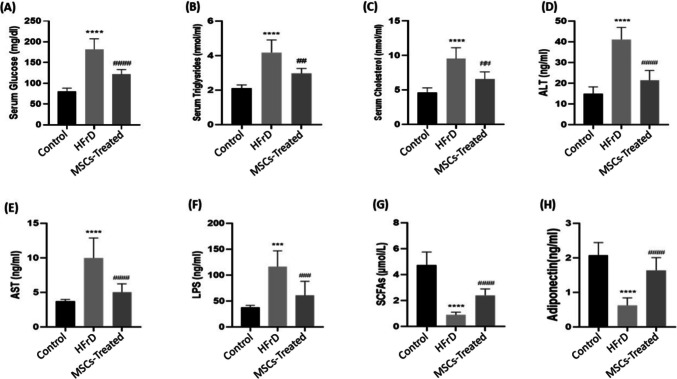
**A**–**H** Effect of MSCs treatment on fasting serum glucose, triglycerides, cholesterol, ALT, AST, LPS, SCFAs, and adiponectin of HFrD rat model. **A** Effect of MSCs on glucose. **B** Effect of MSCs on triglycerides. **C** Effect of MSCs on cholesterol. **D** Effect of MSCs on ALT. **E** Effect of MSCs on AST. **F** Effect of MSCs on LPS. **G** Effect of MSCs on SCFAs. **H** Effect of MSCs on adiponectin: Data are mean ± S.D. *n* = 10 rats per group. ^****^P < 0.0001 vs. control group, ^***^P < 0.001 vs. control group, ^####^*P* < 0.0001 vs. HFrD, ^###^*P* < 0.001 vs. HFrD group, ^##^*P* < 0.01 vs. HFrD group by one-way ANOVA with Tukey’s multiple comparison test. MSCs: mesenchymal stem cells, HFrD: high fructose diet, ALT: alanine transaminase, AST: aspartate aminotransferase, LPS: lipopolysaccharide, SCFAs: short chain fatty acids

Fasting serum glucose level was significantly elevated (*p* < 0.0001) by 2.26 times in rats fed HFrD compared to the control group. Administration of the ADMSCs to the HFrD-fed rats significantly decreased the fasting serum glucose level (*p* < 0.0001) with a percentage change of 33.3% in the stem cell-treated group compared to the HFrD group (Fig. 2A).

### Effect of ADMSCs treatment on lipid profile

There was a significant elevation in serum triglycerides (TGs) and cholesterol levels (*p* < 0.001) by 1.97 and 2.06 times respectively recorded in the HFrD group, compared to the control group. Also, there was a significant reduction of serum TGs and cholesterol levels in the ADMSCs treated group (*p* < 0.01), (with percentage change 29 and 30.95%, respectively), compared to the HFrD group (Fig. 2B, C).

### Effect of ADMSCs treatment on liver functions

Serum alanine transaminase (ALT) and Aspartate Aminotransferase (AST) were elevated significantly (*p* < 0.0001, *p* < 0.001, respectively) by 2.75 and 2.7 times respectively in the HFrD group, compared to the control group. Significant reduction of the serum ALT and AST levels was observed (*p* < 0.0001, *p* < 0.001, respectively) in the ADMSCs treated group (with percentage change 47.87 and 49.7% respectively), compared to the HFrD group (Fig. 2D, E).

### Effect of ADMSCs treatment on serum adiponectin and microbiota metabolites

HFrD group showed a significant elevation (*p* < 0.001) in the serum lipopolysaccharide (LPS) level, by 3.07 times compared to the control group. The treated group showed a significant decrease in serum LPS level (*p* < 0.01), (with percentage change 47.7%) compared to the HFrD group (Fig. 2F). Meanwhile, there was a significant reduction in serum adiponectin and SCFAs levels (*p* < 0.0001) in the HFrD group, (with percentage change 69.9 and 81.2%, respectively), compared to the control group, while a significant elevation of serum adiponectin and SCFAs levels was observed in ADMSCs treated group (*p* < 0.0001) and (*p* < 0.001) respectively by 2.62 and 2.71 times respectively compared to the HFrD group (Fig. 2G, H).

### Effect of ADMSCs treatment on Interleukin-10 (IL-10), superoxide dismutase (SOD), and NADPH oxidase-4 (NOX-4) levels in liver and adipose tissues

HFrD significantly depleted IL-10 levels (*p* < 0.001) as well as SOD enzyme concentrations (*p* < 0.0001) compared to the control group by 60.8% and 62.2% respectively in the liver and by 60.7% and 63.1% respectively in adipose tissue. ADMSCs treatment significantly (*p* < 0.001) restored these depleted levels by 2.28 and 2.02 times respectively in the liver and by 2.25 and 1.81 times respectively in adipose tissue compared to HFrD group Tables 2 and 3.

**Table 2:** Effect of mesenchymal stem cells treatment on liver tissue levels of some related biochemical parameters of high fructose diet-induced insulin resistance rat model

	Control	HFrD	MSCs treated
IL-10 pg/mg	87.5 ± 23.7	34.27 ± 6.958***	78.43 ± 14.99^###^
SOD U/mg	3.046 ± 0.363	1.15 ± 0.17****	2.328 ± 0.463^####^
NOX4 ng/mg	0.814 ± 0.252	2.565 ± 0.427****	1.385 ± 0.366^####^
IRS-1	1.031 ± 0.048	0.202 ± 0.025****	0.661 ± 0.116^####^
AMPK	1.039 ± 0.057	0.159 ± 0.075****	0.631 ± 0.146^####^
SREBP-1C	1.076 ± 0.096	5.07 ± 1.08****	2.276 ± 0.701^####^
LnRNA MALAT-1	1.08 ± 0.099	4.48 ± 1.78**	1.934 ± 0.63^##^

Data are mean ± S.D. *n* = 10 rats per group.**P < 0.01 vs. control group,***P < 0.001 vs. control group,****P < 0.0001 vs. control group,^##^*P* < 0.01 vs. HFrD group,^###^*P* < 0.001 vs. HFrD group,^####^*P* < 0.0001 vs. HFrD group by one-way ANOVA with Tukey’s multiple comparison test. *MSCs* mesenchymal stem cells, *HFrD* high fructose diet, *IL-10* interleukin-10, *SOD* superoxide dismutase, *NOX-4* NADPH oxidase-4, *IRS-1* insulin receptor substrate-1, *AMPK* AMP-activated protein kinase, *SREBP-1C* sterol regulatory element-binding protein-1, *LnRNA MALAT-1* long non-coding RNA of metastasis-associated lung carcinoma transcript-1

**Table 3:** Effect of MSCs treatment on adipose tissue levels of some related biochemical parameters of high fructose diet-induced insulin resistance rat model

	Control	HFrD	MSCs treated
IL-10 pg/mg	87. 33 ± 20.62	32.54 ± 11.98****	73.2 ± 13.29^###^
SOD U/mg	3.01 ± 0.26	1.111 ± 0.219****	2.015 ± 0.326^####^
NOX4 ng/mg	0.579 ± 0.127	2.159 ± 0.479****	1.161 ± 0.232^####^
AMPK	1.042 ± 0.0519	0.187 ± 0.037****	0.728 ± 0.206^####^
IRS-1	1.026 ± 0.0544	0.237 ± 0.059***	0.744 ± 0.34^##^

Data are mean ± S.D. *n* = 10 rats per group.**P < 0.01 vs. control group,***P < 0.001 vs. control group, ****P < 0.0001 vs. control group, ^##^*P* < 0.01 vs. HFrD group, ^###^*P* < 0.001 vs. HFD group, ^####^*P* < 0.0001 vs. HFrD group by one-way ANOVA with Tukey’s multiple comparison test. *MSCs* mesenchymal stem cells, *HFD* high fructose diet, *IL-10* interleukin-10, *SOD* superoxide dismutase, *NOX-4* NADPH oxidase-4, *IRS-1* insulin receptor substrate-1, *AMPK* AMP-activated protein kinase

HFrD significantly increased NOX-4 (*p* < 0.0001) compared to the control group by 3.15 and 3.72 times in liver and adipose tissue respectively. Meanwhile, ADMSCs treatment significantly (*p* < 0.0001) reduced the level of NOX-4 compared to the HFrD group by 46% in both liver and adipose tissue Tables 2 and 3.

### Effect of ADMSCs on AMP-activated protein kinase (AMPK) and insulin receptor substrate-1 (IRS-1) levels in liver and adipose tissues

**Fig. 3:**
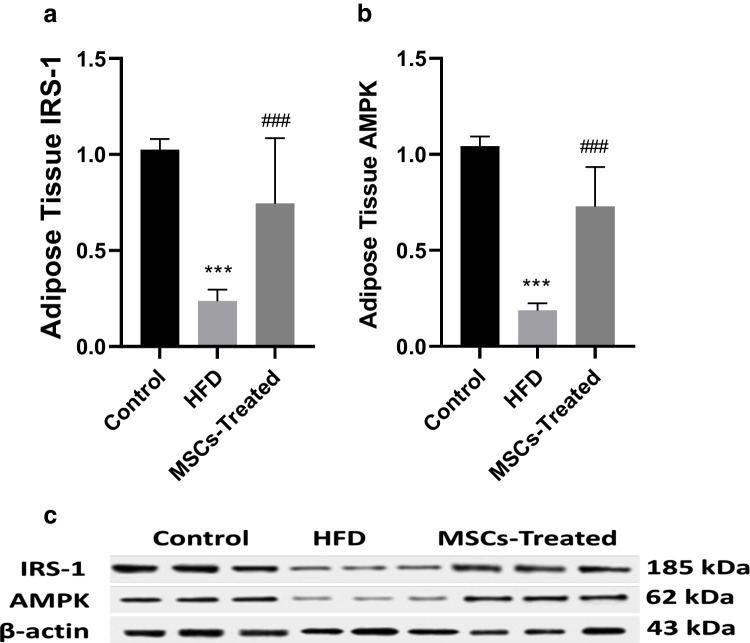
**A**–**C** Effect of MSCs treatment on adipose tissue level of IRS-1 and AMPK of HFrD rat model. **A** Effect of MSCs on IRS-1. **B** Effect of MSCs on AMPK. **C** IRS-1 and AMPK were determined by Western blot: data are mean ± S.D. *n* = 10 rats per group. ^****^P < 0.0001 vs. control group, ^***^P < 0.001 vs. control group, ^####^*P* < 0.0001 vs. HFrD, ^###^*P* < 0.001 vs. HFrD group by one-way ANOVA with Tukey’s multiple comparison test. MSCs: mesenchymal stem cells, HFrD: high fructose diet, IRS-1: insulin receptor substrate-1, AMPK: AMP-activated protein kinase

**Fig. 4:**
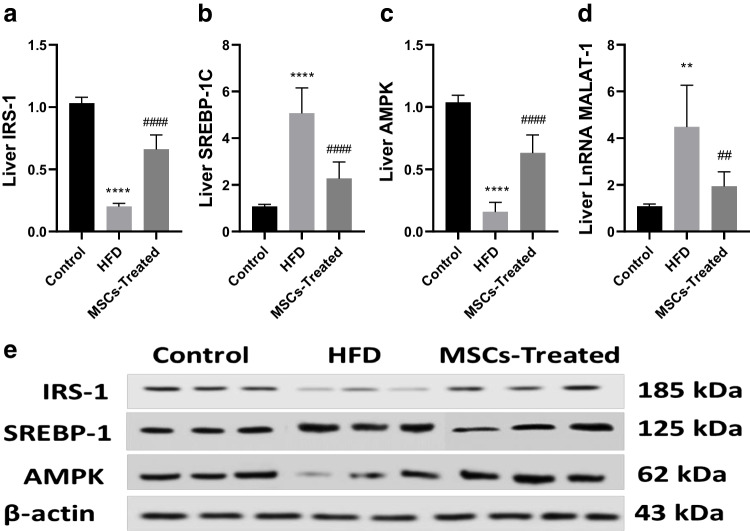
**A**–**E** Effect of MSCs treatment on liver tissue level of IRS-1, SREBP-1C, AMPK, liver LnRNA MALAT-1 of HFrD rat model. **A** Effect of MSCs on IRS-1. **B** Effect of MSCs on SREBP-1C. **C** Effect of MSCs on AMPK. **D** Effect of MSCs on LnRNA MALAT-1. **E** IRS-1, SREBP-1C, AMPK, and LnRNA MALAT-1 were determined by Western blot: data are mean ± S.D. *n* = 10 rats per group. ^****^P < 0.001 vs. control group, ^####^*P* < 0.001 vs. HFrD group by one-way ANOVA with Tukey’s multiple comparison test. MSCs: mesenchymal stem cells, HFrD: high fructose diet, IRS-1: insulin receptor substrate-1, SREBP-1C: sterol regulatory element-binding protein-1, AMPK: AMP-activated protein kinase, LnRNA MALAT-1: long non-coding RNA of metastasis-associated lung carcinoma transcript-1

The protein expression of AMPK and IRS-1 in the liver and adipose tissues in the HFrD group was obviously downregulated compared with the control group (*p* < 0.001) with % changes of 84.7% and 80.4% respectively in the liver plus 82.05 and 76.9% respectively in adipose tissue. Moreover, their expression was evidently enhanced after treatment with ADMSCs by 3.96 and 3.27 times respectively in the liver and by 3.89 and 3.14 times respectively in adipose tissue compared with the HFrD group (*P* < 0.01) (Figs. 3 and 4).

### Effect of ADMSCs treatment on sterol regulatory element-binding protein-1 (SREBP-1C) protein expression and metastasis-associated lung carcinoma transcript-1 (MALAT-1) gene expression

We observed significantly higher SREBP-1C (*p* < 0.0001) and MALAT-1 (*P* < 0.01) expression levels in the HFrD group as compared to the control group by 4.71 and 4.14 times respectively. Compared to the HFrD group, both SREBP-1C and MALAT-1 showed significantly lower expression by 55.1 and 56.8% in the MSCs-treated group ((*P* < 0.0001) and (*P* < 0.01) respectively) (Fig. 4).

## Discussion

In the last decades, one of the most pivotal adjustments in the food industry was the high incorporation of fructose in multiple products. Consequently, many adverse effects have been spotlighted.

Mesenchymal stem cells (MSCs) are multipotent cells that reside in adult tissues and can differentiate into many cell types. Recently, their multi-potentiality and peculiar ability to self-renew have given them a bright approach to being involved in the treatment of different diseases. Several clinical trials have been performed to assess the potency of MSCs in ameliorating several diseases (Tsuchiya et al. 2019; Shin et al. 2020).

Because of their many advantages, adipose-derived mesenchymal stem cells (ADMSCs) are becoming growing in popularity in the field of regenerative medicine. They are readily and plentifully available from adipose tissue, which is usually extracted using simple techniques like liposuction (Ahmadieh-Yazdi et al. 2024). The yield of stem cells per unit of tissue from ADMSCs is larger than that of stem cells from other sources, such as bone marrow (Via et al. 2012). ADMSCs have potent anti-inflammatory and immunomodulatory qualities that make them useful in the treatment of inflammatory and autoimmune illnesses (Ortiz et al. 2023). They also lessen the risk of immunological rejection in transplant recipients (Farhana et al. 2023). Their potential is further enhanced by their capacity to release growth factors and cytokines, which facilitate tissue healing and the development of new blood vessels (Mazini et al. 2020). ADMSCs are more widely accepted in therapeutic settings because, in contrast to embryonic stem cells, they pose fewer ethical questions (Robertson 2001). However, ADMSCs efficacy can be impacted by the age and health of the donor, which can also affect the quality and properties of the cells (Choudhery et al. 2014). Although they have the ability to differentiate into several cell types, their range is somewhat more limited than that of other stem cells, such as induced pluripotent stem cells (Dupuis and Oltra 2021). Potential safety concerns include the possibility of unintentional cell proliferation or tumor formation (Liang et al. 2021). Ensuring consistent quality and safety in clinical applications is also a difficulty (Fernández-Santos et al. 2022). Additionally, depending on the therapeutic objective, ADMSCs have a tendency to favor fat cell development, which is not always desired (Liu et al. 2024).

This study aimed to elucidate the protective effect of ADMSCs against the deleterious effect of a high fructose diet on the disturbance of the physiological functions of various body organs, primarily liver dysfunction and genesis of insulin resistance. Administration of a high fructose diet caused a significant elevation of body weight compared to control rats. Furthermore, rats that received a high fructose diet significantly had a high serum fasting blood glucose level. Ondee et al. reported a significant weight gain in rats with a high fructose diet in the experiment. Also, they observed an elevation of fasting blood glucose after fructose administration (Ondee et al. 2023). While Ortega-Pérez et al. observed that rats fed with a high fructose diet for 13 weeks, showed a significant increase in body weight between the 6th (15.5%) and 13th (18.0%) week as compared to the control group (Ortega-Pérez et al. 2022).

Cholesterol and TG levels significantly increased in rats who received HFrD compared to control rats. These outcomes are consistent with preceding studies. Ondee et al. reported that rats with a high fructose diet showed a significant increase in total cholesterol, triglycerides, and LDL (Ondee et al. 2023). Also, Park et al. revealed that the toxic effect of a high fructose diet on lipid profile was demonstrated by an increase in TG, TC, glucose, and leptin levels (Park et al. 2020). Prasartthong et al. found that blood glucose, insulin, cholesterol, and triglycerides levels were significantly higher, while HDL-C levels were markedly lower in the high fructose diet group than in the control group (Prasartthong et al. 2022). On the other hand, rats that received ADMSCs distinguished a reduction in body weight along with a decrease in serum TG, cholesterol, and blood glucose levels compared to the HFrD group. These coincided with Wang et al. who demonstrated the ability of the ADMSCs to decrease body weight besides Cholesterol and TGs levels in rats that developed obesity and dyslipidemia (Wang et al. 2019).

Fructose goes to the liver, where it is converted to pyruvate via a glycolytic pathway; then, in mitochondria, fructose is metabolized to acetyl-CoA through the tricarboxylic acid (TCA) cycle. Excess acetyl-CoA moves to the cytoplasm, which converts to citrate, which is essential for de novo lipogenesis (Cho et al. 2021).

In the current study, a significant increase in serum ALT and AST was detected in the HFrD group as compared to the control group. This is consistent with previous findings in the study of Park et al. who measured the activities of serum ALT, AST, and ALP to evaluate liver function and damage and found that serum ALT, AST, and ALP levels were significantly higher in the high fructose diet group than in the normal control group (Park et al. 2020). Also, Wang et al. observed that serum AST and ALT did not change significantly after 4 weeks of high fructose diet but increased markedly at 8 and 12 weeks in the high fructose diet group (Wang et al. 2021). In addition, Shu et al. reported that the levels of ALT and AST also increased significantly in the high fructose diet group (Shu et al. 2022).

On the contrary, ADMSCs injection established a decrease in the serum ALT and AST in comparison to rats received a high fructose diet, which concurred with Yu et al. who demonstrated that levels of ALT and AST plus cholesterol, TGs, and LDL-C significantly decreased in diabetic rats after injection of ADMSCs (Yu et al. 2019). Likewise, ADMSCs diminished AST and ALT levels and improved liver fibrosis after massive deterioration by CCl4 that induced marked liver dysfunction (Liao et al. 2020).

To explore the mechanism by which fructose disrupted physiological functions and generated liver damage, we examined the elaboration of oxidative stress by determining hepatic SOD and NOX4 levels. Administration of high fructose diet resulted in a significant decrease of SOD associated with a significant increase of NOX4 relative to control rats. Otherwise, ADMSCs avoided these effects. These are consistent with Park et al., who confirmed an incredible decline of CAT, GPx, and SOD in rats after feeding high fructose (Park et al. 2020).

The NOX family has been implicated as a valuable concern for the progression of reactive oxygen species. Certainly, NOX4 creates enormous amounts of hydrogen peroxide (H_2_O_2_) that do not need GTPase Rac to be activated. Also, NOX4 can be in contact with cell membrane internal membranes, allowing the production of H_2_O_2_ (Herranz-Itúrbide et al. 2021). Additionally, NOX oxidizes oxygen to the superoxide anion via NADPH use. Also, NOX is competent in activating xanthine oxidoreductase (XO), which encourages the hydroxylation of hypoxanthine to xanthine and then xanthine to uric acid. These are the primary roots for generating intracellular hepatic ROS (Muriel et al. 2021).

The antioxidant properties of ADMSCs were proved in various studies. El-Sawah et al. detected that ADMSCs reversed the elevation of MDA, ROS, and XO and the decrease of GSH, SOD, CAT, GST, TAC, and HO-1 in the pancreas of diabetic rats (El-Sawah et al. 2022). Moreover, Goudarzi et al. estimated the ability of ADMSCs to improve chronic prostatitis through augmentation of SOD and GPx accompanied by suppression of MDA (Goudarzi et al. 2021).

In the present study, IL-10 level was observed to be significantly decreased in fructose-feeding rats compared to the control group, while ADMSCs injection altered this result. Cheng et al. demonstrated that high fructose contributed to the inflammation response exhibited by a marked increase of IL-1β, IL-6, and TNF-α plus a reduction of IL-10 (Cheng et al. 2022). Similar results were observed by Casagrande et al. who recorded that the high fructose established a deficient IL-10 level (Casagrande et al. 2019).

The anti-inflammatory effect of adipose tissue mesenchymal stem cells was demonstrated and confirmed by previous studies. Hashemi et al. who assessed the concentration of inflammatory and anti-inflammatory cytokines in supernatant of splenocytes after adipose tissue mesenchymal stem cells administration, found higher levels of IL-10 in mesenchymal stem cells treated rats compared to diabetic rats (Hashemi et al. 2020). Also, Yu et al. revealed a lower expression of genes encoding molecules typically linked to inflammation (TNF-α, IL-1β) or fibrosis (TGF-β) and a higher expression of genes encoding anti-inflammatory molecules (IL-10) in the adipose tissue mesenchymal stem cells treated group (Yu et al. 2019).

As significant incorporation was found between gut microbiota and adjustment of many physiological functions including generation of metabolic disorders, we examined microbiota markers, including SCFAs and LPS. We noticed that high fructose ingestion resulted in a significant decrease in SCFAs, but LPS showed a significant increase compared to controls. ADMSCs inverted these findings and validated the participation of ADMSCs in the reinforcement of microbiota attributes. Tan et al. found that the levels of 25 SCFAs in the high-fructose group were significantly lower compared to the control group. (Tan et al. 2021). Meanwhile, Coutiño-Hernández et al. found that the group fed with a high fructose diet showed significantly higher circulating levels of LPS compared to the control group (Coutiño-Hernández et al. 2022). On the other hand, Sun et al. showed that ADMSCs increased the abundance of gut microbiota, which can regulate the thickness of the intestinal mucosa, so as to maintain the intestinal barrier and provide a protective effect decreasing the circulating levels of LPS (Sun et al. 2020).

Hepatic lipogenesis induced by fructose seems to be related to acetate derived from microbiota (Zhao et al. 2020). Moreover, the production of LPS is implied to be primarily involved in fructose-generated liver steatosis (Todoric et al. 2020; Febbraio and Karin 2021). Also, fructose consumption is attributed to disturbances of microbiota structure with the enhancement of the development of obesity (Wang et al. 2022). On the contrary, SCFA metabolites preserve intestinal stability through the activation of Th1 cells plus IL-10 secretion (Sun et al. 2018).

As reported before, the measurement of adiponectin levels recorded a significant decrease in the high fructose group compared to control rats. Park et al. noticed a significant decrease in adiponectin levels experienced by high fructose diet-fed rats. Also, Gumede et al. detected a significant decrease in adiponectin levels in rats fed a high fructose diet as compared to the control group (Gumede et al. 2020).

However, rats who received ADMSCs showed a significant increase as recorded formerly. Yang et al. found that adiponectin secretion is enhanced by ADMSCs. Also, they suggested that ADMSCs increased the expression of both adiponectin receptors 1 and 2 (Yang et al. 2019).

Adiponectin has a powerful anti-inflammatory character and plays a significant role in preventing insulin resistance with a decrease in the risk for the development of obesity and metabolic syndromes. Adiponectin stimulates AMPK pathways through adiponectin receptors, enhancing the oxidation of fatty acid and muscle glucose uptake, plus hepatic gluconeogenesis inhibition (Li et al. 2019).

To investigate the molecular mechanisms underlying liver dysfunction and metabolic disturbance induced by a high fructose diet, the expression of lipogenesis-related genes was examined. Our findings demonstrated a significant downregulation of AMPK and IRS-1 in both liver and adipose tissue while establishing upregulation of SREBP-1C and MALAT-1 in the high fructose diet group, while administration of ADMSCs abolished these findings. Recently, SREBP-1c levels were observed to be significantly upregulated in the livers of high fructose diet rats compared to normal control rats as well as a significant downregulation of the AMPK signaling pathway (Park et al. 2020).

In the study of Song et al. mice treated with mesenchymal stem cells showed a promoted phosphorylation of the AMPK signaling pathway with subsequent enhancement of their activity (Song et al. 2023). Wang et al. also found that the expression of SREBP-1c and fatty acid synthase (FAS) was significantly downregulated in the ADMSCs group (Wang et al. 2019). Concerning the therapeutic effects of ADMSCs on the insulin resistance model, the expression levels of IRS-1 and GLUT4 were examined by qRT-PCR and western blot analysis in the study of Kim and colleagues. Both IRS-1 and GLUT-4 mRNA expression levels were significantly increased after ADMSCs administration (Kim et al. 2018).

AMPK has a crucial impact on the maintenance of energy stability, mainly glycolysis and fatty acid oxidation. AMPK is demonstrated to be activated through binding to CircACC1, a circRNA derived from the human acetyl-CoA carboxylase 1* (*ACC1*)* gene, thus inhibiting lipogenesis. On the contrary, SREBP-1c is essential for hepatic and adipose tissue lipogenesis by enhancing fatty acid synthase expression (Liou et al. 2019). Furthermore, MALAT1 is markedly concerning with abnormal cholesterol and fatty acid synthesis with consequent lipid accumulation (Lu et al. 2022). Furthermore, the knockdown of MALAT-1 is associated with stimulation of autophagy and apoptosis of granulosa cells, thereby reducing the proliferation of endometriosis via upregulation of AMPK expression. (Liu et al. 2021).

## Limitations

This study may have some limitations that need to be further investigated to get a full picture of physiological function disturbances that occur from a high fructose diet. At the same time to make a complete figure about the beneficial effects of stem cells and further studies to detect therapeutic approaches of ADMSCs. Some of these limitations include the performance of a stool culture to identify the composition of gut microbiota and the effects of high fructose on the alternation of microbiota distribution. Moreover, the duration of the study may limit the capacity to fully understand the long-term effects of ADMSC treatment on metabolic disorders resulting from different mechanisms. Furthermore, the application of preclinical findings to clinical use has multiple challenges, including safety and efficacy.

## Conclusion

According to the findings of this study, ADMSCs are anticipated to be a promising agent against metabolic hazards induced by a high fructose diet, particularly insulin resistance. This achievement may be related to antioxidants and anti-inflammatory properties, besides metabolic pathways management. For example, enhancement of adiponectin secretion, upregulation of AMPK/IRS-1, and inhibition of MALAT-1 and SREBP-1C.

## Data Availability

The authors confirmed that the data supporting the results of the current study, and its supplementary materials are available from the corresponding author on reasonable request.
